# The relationship between interpersonal security and social media dependence: a moderated mediation model

**DOI:** 10.3389/fpsyt.2025.1536539

**Published:** 2025-02-26

**Authors:** Jinglin Li, Jiajia Tan, Shang Zhang, Haihong Wang, Xinfa Yi

**Affiliations:** ^1^ Key Laboratory of Modern Teaching Technology, Ministry of Education, Shaanxi Normal University, Xi'an, China; ^2^ Curriculum and Management Major, Department of Education, Shaanxi Normal University, Xi'an, China; ^3^ Psychological Health Education Center, Student Affairs Department, Northwest University, Xi'an, China; ^4^ Beijing Normal University Collaborative Innovation Center for Quality Inspection of Basic Education Shaanxi Normal University Branch, Xi'an, China

**Keywords:** college students, interpersonal security, negative rumination, sibling condition, social media dependence

## Abstract

**Background:**

Interpersonal security is an important psychological factor influencing social media use. However, little is known about the mediating and moderating mechanisms linking Interpersonal security and social media dependence.

**Objective:**

The present study explored the mediating role of negative rumination between interpersonal safety and social media dependence, as well as cohort differences in sibling conditions as moderators.

**Methods:**

A total of 986 college students were surveyed using a cross-sectional design. Participants completed the Interpersonal Security Questionnaire, the Social Media Dependence Scale, and the Negative Rumination Scale.

**Results:**

The results showed that a significant interrelationship between interpersonal security, negative rumination, and social media dependence. In addition, the role of negative rumination as a mediator of interpersonal security and social media dependence was supported, and the mediating effects were different between the only-child and non-only-child cohorts.

**Conclusions:**

Findings of the study provide a psychological basis for the treatment of social media dependence behavior in college students, with the aim of increasing their interpersonal security and reducing their dependence on social media.

## 1 Introduction

Social media addiction has become a global phenomenon, especially among young adults, and is detrimental to their physical and mental health (1). According to the Social Networking and Empowerment Research Report, 73% of young adults check social software every 15 minutes, 71.3% often open social software even at work or in a meeting, and 84.4% feel anxious if their mobile phones can’t connect to the internet (2, 3). College students with social media dependence suffer from loneliness, anxiety, depression, sleep disorders, stress, burnout, and decreased academic performance (4, 5).

Psychological factors such as self-esteem, anxiety and depression, instant gratification, and psychological needs can influence social media addiction (6, 7). Unlike other psychological factors, interpersonal safety, which refers to a sense of security and trust in others, is also an important component of security and a central factor in the development of interpersonal relationships (8, 9). Maslow argues that when physiological needs are largely met, individuals increasingly seek safety, stability and security in their environment (10, 11). Based on self-determination theory, the need for relationships is one of the most basic psychological needs of individuals who have a social tendency to be in touch with others to gain a sense of security (12, 13). The advent of social media offers a new way of developing and maintaining relationships. The psychological decompensation hypothesis states that when normal psychological development is hindered by internal and external factors, individuals turn to addictive behaviors as a way to compensate for their psychological deficits (14, 15).

In the current era, social media dependence and interpersonal security are not deeply explored. Internet usage may be more common among people who are interpersonally insecure. They may seek emotional support and a sense of belonging through social media, online gaming, or virtual communities (16). Further, there is a significant negative correlation between interpersonal security and behavioral addiction, and individuals with a greater sense of security are less likely to develop Internet dependence or addictive behaviors as a way of escaping the reality of social anxiety or loneliness (17). There is also evidence that the Internet can enhance real-life social relationships for those with a high level of interpersonal security. For example, keeping in touch with friends and family through social media (18, 19). Therefore, exploring the relationship between interpersonal security and social media dependence and its mechanism of action can provide important insights for the effective prevention and intervention of social media dependence among young adults.

Previous study showed that Individuals with low self-security are prone to negative rumination when confronted with a threat they cannot cope with (20). Negative rumination is a cognitive pattern characterized by repeatedly dwelling on the cause and effect of a negative event without taking any action to address the issue (21, 22). On the other hand, negative rumination can be a trigger for an individual’s addiction to social. Based on Interaction of Person Affect Cognition Execution (I-PACE) theoretical model, individuals may use the Internet to escape negative emotions and obtain temporary pleasure, and rumination can exacerbate this behavior, ultimately leading to internet addiction. Similar to this result, a study showed that individuals with depression or anxiety were more likely to ruminate and increase their smartphone use. Rumination is one of the important mechanisms to explain smartphone dependence (23). Regarding the relationship between interpersonal security, social media dependence, and rumination, only one study found that rumination can cause excessive reassurance-seeking behavior by triggering habitual and constant checking of one’s smartphone for social-related notifications (24). Given that this is a case study and cannot be generalized, the relationship deserves further investigation.

The implementation of the three-child policy in China has brought multi-child families back into an important part of society. Research suggests that having siblings can provide positive feedback and a greater sense of security (25). Some researchers have argued that compared with the non-only-child group, only child tend to be more selfish, lonely, narcissistic, and uncooperative due to excessive attention and praise from family members and are weak in social interaction and agreeableness (26). However, some studies indicating that only children have certain advantages, such as better academic achievement, leadership, and self-concept in China (27). In addition, only children have no obvious disadvantages in social skills and self-concept (28). Previous research has shown that being an only child affects online media use. The frequency and duration of online media use were higher among only children compared to non-only children. The reason for this phenomenon may be that only children are more lonely at home and therefore rely more on online social media to seek interaction with others, especially with family members (29). However, some researchers have also suggested that compared with non-only children, only-child counterparts will be immune to problematic internet use (PIU) when they show a preference for online social interaction (30).

The aims of this study were: 1. to explore the relationship between interpersonal security, social media addiction and negative rumination; and 2. to explore the effect of having or not having siblings on the relationships between interpersonal security and social media addiction. We hypothesis that individuals with interpersonal insecurity may experience negative emotions through rumination, leading them to seek refuge or emotional support on the internet, particularly social media, and the presence of siblings may modulate this relationship.

## 2 Methods

### 2.1 Participants

This study has obtained ethical review approval from the Ethics Committee of the Key Laboratory of Modern Teaching Technology, Ministry of Education, Shaanxi Normal University, with approval number: L20230410-02. Convenience sampling was used to select 1050 students from a university in Xi’an, Shaanxi Province, to complete demographic interviews and related questionnaires. Inclusion criteria were (1): participants were full-time undergraduate students from freshmen to seniors, (2) participants voluntarily participated in the study, and (3) participants were able to comprehend and independently complete the questionnaires. Exclusion criteria were: (1) participants who did not complete the questionnaire, (2) those with substantial missing data, (3) participants who exhibited patterned response behaviors (e.g., excessive selection of the same option), and (4) participants who were part-time students or no longer enrolled. After excluding invalid responses, 986 valid questionnaires were collected (male: 349; female: 637, only-child: 409; non-only-child: 577, *M* = 21.38, *SD* = .893), giving an effective rate of 93.90%.

### 2.2 Materials

The Interpersonal Security Questionnaire, the Social Media Dependence Scale and the Negative Rumination Scale were used to measure interpersonal security, social media dependence and rumination, respectively. The data were collected using cross-sectional data and self-report scales. The Interpersonal Security Questionnaire was adapted from the Security-Insecurity Questionnaire (S-I) developed by Maslow et al. (11), which was revised and translated into the Chinese version by Cao et al. (31). Permission to use this scale has been obtained. It consists of 18 items with 4 second-order factors: Emotional Harmony, Optimism and Cheerfulness, Respect and Love, and Happiness and Warmth. An example item is “Generally speaking, I am an optimist”. Each item consists of 3 options: “Yes” “No” and “Unclear”, where “No” and “Unclear” are scored as “0”, while “Yes” is scored as “1”. A higher arithmetic mean score indicates greater interpersonal security. The total Cronbach’s alpha coefficient for this scale is.934, and the Cronbach’s alpha coefficients for the subscales are.798,.862,.813, and.909, respectively.

The Bergen Social Media Addiction Scale (BSMAS) consists of 6 items and is rated on a 5-point Likert scale, where 1 indicates “very little” and 5 indicates “very much”. An example item is “Do you spent a lot of time thinking about social media and planning the use of social media?” Higher scores indicating higher levels of addiction. The overall Cronbach’s alpha coefficient for this scale is.887.

The Positive and Negative Co-Rumination Scale developed by Yang et al. (32) was adopted and consists of 13 items with 3 second-order factors: Suppress Happiness, Self-Denial and Negative Attributions. Each item is scored on a scale ranging from 1 (never) to 4 (always). An example item is “Think Happy days do not last long.” Higher scores indicate higher levels of negative rumination. The overall Cronbach’s alpha coefficient for this scale is.915, and the Cronbach’s alpha coefficients for the subscales are.873,.845, and.871, respectively.

### 2.3 Statistical analyses

Descriptive statistics and correlation analysis were conducted using software. AMOS 21.0 was used for the mediating effect tests and group effect analyses, and 2000 bootstrap samples were selected to estimate the 95% confidence intervals of the mediating effects by using the deviation-corrected percentile bootstrap method.

### 2.4 Test of common method bias

A Harman’s single-factor test was used to test for common method bias. The results showed that there were six factors with eigenvalues greater than 1, and the variance explained by the variance of the first factor was 30.22%, which was less than 40%, indicating that the variables in this study do not exhibit common method bias.

## 3 Results

### 3.1 The relationships between interpersonal security, social media dependence and negative rumination in only-child group and non-only-child group

**Figure 1:**
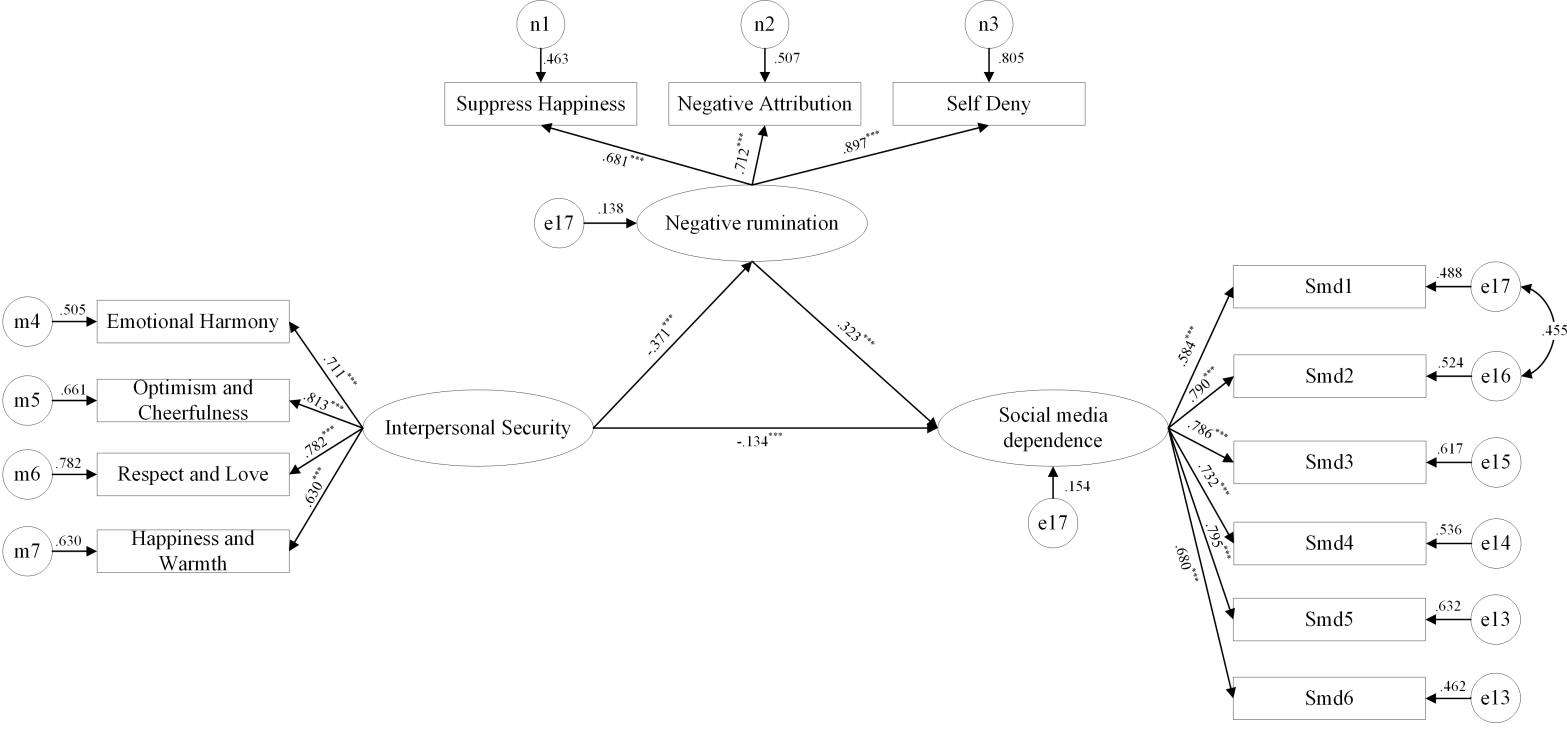
The mediating role of negative rumination between interpersonal security and social media dependence.

Pearson correlation analyses were conducted t for the variables of interest in the only-child and non-only-child groups respectively only-child. The results showed that (see **Table 1**) interpersonal security, social media dependence and negative rumination were significantly correlated in both the only-child group and non-only-child group. Regression analysis of the dependent variable “social media dependence” with interpersonal security as the independent variable revealed that interpersonal security had a significant negatively predicted social media dependence(*β* = -0.167, *t* =-4.074, *ρ* < 0.001). The higher the interpersonal security, the lower the college students’ dependence on social media. In order to further explore the mechanism of interpersonal security on social media dependence, the mediating role of negative rumination thinking between interpersonal security and social media dependence was examined through structural equation modeling using AMOS24.0 software. First, an unmediated model (M1) was constructed with interpersonal security as the independent variable and social media dependence as the dependent variable, and the results showed a good fit (M1: *χ^2^/df* = 4.696, *CFI* = .977, *GFI* = .969, *AGFI* = .948, *TLI* = .968, *NFI* = .971, *RMSEA* = .061), with interpersonal security significantly negatively predicting social media dependence (*β* = -.253, *p <0*. 001). Then, negative rumination was introduced into M1 to construct the mediation model (M2a), which showed that the fit was general and that the model required further modification (*χ^2^/df* = 6.782, *CFI* = .945, *GFI* = .934, *TLI* = .931, *NFI* = .936, *RMSEA* = .077). Next, the covariance of the error variables in the same factor were added based on the information in the “Modification Indices”, (see **Figure 1**), and the corrected model (M2b) showed a statistically significant fit (M2b: *χ^2^/df* = 4.449, *CFI* = .968, *GFI* = .959, *TLI* = .959, *NFI* = .959, *RMSEA* = .059).

**Table 1:** Correlation analysis of interpersonal security, negative rumination and social media dependence among only children and non-only children (n = 986).

	1	2	3	4	5	6	7	8	9	10
Social Media Dependence	1	.29^***^	.13**	.33^***^	.30^***^	-.17^***^	-.13^***^	-.14^***^	-.13^***^	-.17^***^
Negative Rumination	.31^***^	1	.84^***^	.77^***^	.89^***^	-.34^***^	-.33^***^	-.29^***^	-.27^***^	-.28^***^
Suppress Happiness	.22^***^	.89^***^	1	.43^***^	.57^***^	-.24^***^	-.23^***^	-.19^***^	-.20^***^	-.18^***^
Self-denial	.31^***^	.75^***^	.47^***^	1	.64^***^	-.38^***^	-.35^***^	-.34^***^	-.29^***^	-.31^***^
Negative Attributions	.31^***^	.92^***^	.70^***^	.63^***^	1	-.28^***^	-.27^***^	-.23^***^	-.22^***^	-.23^***^
Interpersonal Security	-.29^***^	-.35^***^	-.28^***^	-.39^***^	-.29^***^	1	.80^***^	.87^***^	.89^***^	.84^***^
Emotional Harmony	-.24^***^	-.25^***^	-.19^***^	-.29^***^	-.20^***^	.79^***^	1	.58^***^	.65^***^	.51^***^
Optimism and Cheerfulness	-.25^***^	-.35^***^	-.27^***^	-.38^***^	-.28^***^	.88^***^	.57^***^	1	.69^***^	.66^***^
Respect and Love	-.27^***^	-.30^***^	-.24^***^	-.33^***^	-.25^***^	.90^***^	.66^***^	.72^***^	1	.69^***^
Happiness and Warmth	-.23^***^	-.31^***^	-.25^***^	-.33^***^	-.25^***^	.85^***^	.50^***^	.68^***^	.72^***^	1

Below the slash is the correlation coefficient for the only child group, and above the slash is the group correlation coefficient for the non-only child group. **p < 0.01, ***p < 0.001, which is the same as below.

The results showed (**Figure 1**) that a significance test of the mediation effect using bootstrapping with a self-sampling size of 2000 revealed a 95% confidence interval of (-.685, -.156) between interpersonal security and social media dependence that did not include 0, indicating a significant direct effect. The 95% confidence interval for negative rumination between interpersonal security and social media dependence was (-.532, -.236), which did not include 0. The mediation effect was significant with a value of -.363, accounting for 47.33% of the total effect. The partial mediating effect of negative rumination on the relationship between interpersonal security and social media dependence was significant. Specifically, interpersonal security significantly and negatively predicted social media dependence (*β* = -.134, *p* < 0.001). Interpersonal security significantly and negatively predicted negative rumination thinking (*β* = -.371, *p* < 0.001), and negative rumination thinking significantly and positively predicted social media dependence (*β* = .323, *p* < 0.001).

### 3.2 Differences in the mediating role of negative rumination in the only-child and non-only-child groups

**Figure 2:**
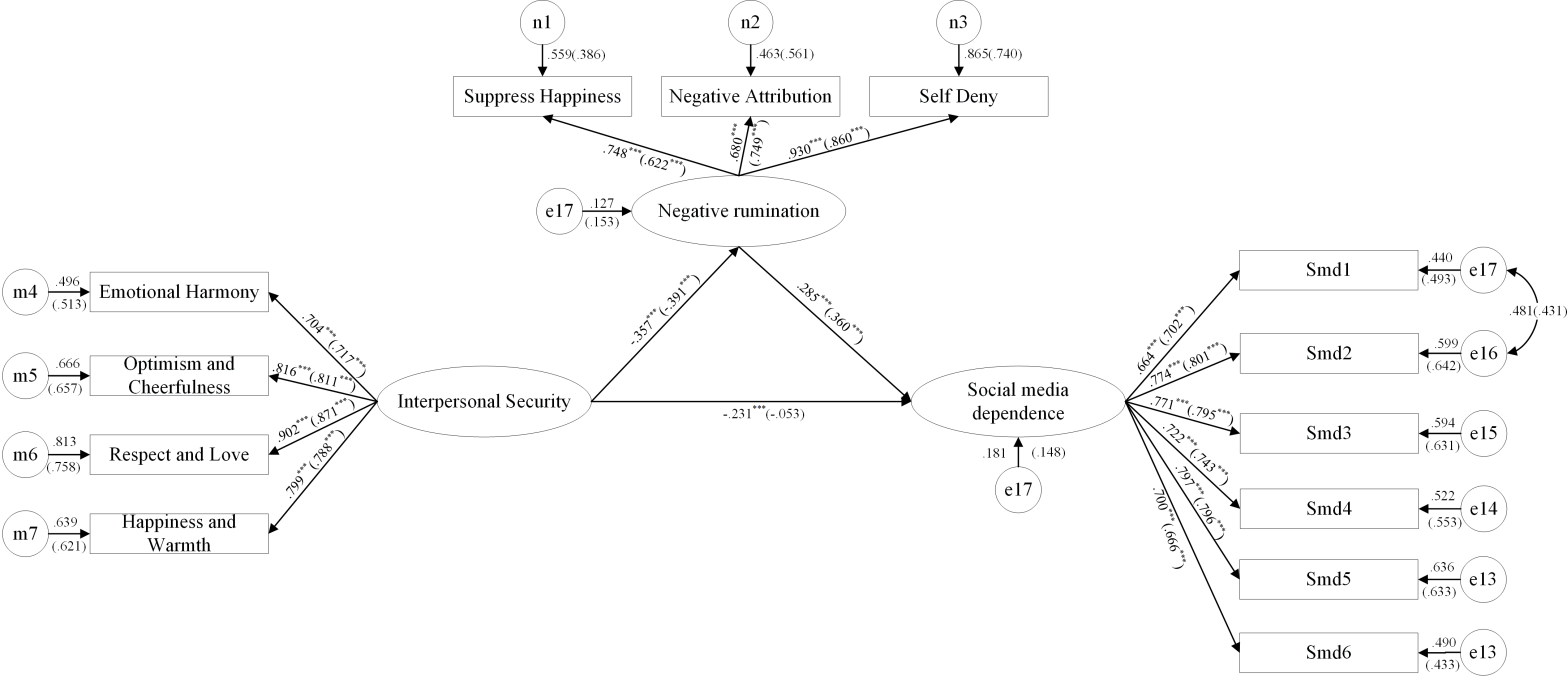
The mediating role of negative rumination between interpersonal security and social media dependence for only children and non-only children. The results for only children are outside the parentheses, and those for non-only children are inside the parentheses.

To test whether the mediating effect of negative rumination on interpersonal security and social media dependence differed between the only-child group and non-only-child groups, a multi-group model was constructed based on the mediation model (M2b), with only-children and non-only-children as group variables for testing (**Figure 2**). Both the only-child cohort model (*χ^2^/df* = 2.581, *CFI* = .965, *GFI* = .945, *TLI* = .956, *NFI* = .945, *RMSEA* = .062) and the non-only-child cohort model (*χ^2^/df* = 2.634, *CFI* = .973, *GFI* = .959, *TLI* = .966, *NFI* = .958, *RMSEA* = .053) all achieved acceptable levels of fit, indicating to the need for cross-cohort comparisons. On this basis, multicluster comparison in structural equation modeling was used to set the equivalent model, and the unrestricted model and several restricted models were compared and examined, with the order of restriction intensity from smallest to largest being the unrestricted model, the measurement coefficient model, the structural coefficient model, the structural covariance model, and the structural residual model (see **Table 2**). The results showed that the differences in the fitting indices (ΔCFI, ΔTLI, and ΔRMSEA) between the two pairs of models were less than.01, indicating that the equivalent models were valid and that the mediation model of negative rumination had the same significance and potential structure for only children and non-only children. The path coefficients of the only-child group and the non-only-child group were further compared and the critical ratio value was used for analysis. When the absolute value of the critical ratio was greater than 1.96, there was a difference in one of the path coefficients between the two groups. There was a significant difference in interpersonal security—the social media dependence path—between the only-child group and the non-only-child group. Specifically, the coefficient of the interpersonal security-social media dependence path in the only-child group was significantly greater than that in the non-only-child group. The bootstrap method was used to test the mediating effect of negative rumination between only children and non-only children groups. The results showed that the 95% confidence intervals of the mediating effect of negative rumination between interpersonal security and social media dependence were (-.614, -.149) and (-.666, -.236) for only children and non-only children, respectively. That is, the mediating effect of negative rumination was significant in both the only-child group and the non-only-child group. Further analyses show that the mediated effect size of negative rumination among only children was -.320, accounting for 30.6% of the total effect size. The mediating effect size of negative rumination in the non-only children was -.414, accounting for 72.5% of the total effect size. The mediating effect of negative rumination was significantly higher in the non-only-child group than that in the only-child group.

**Table 2:** The fitting index of the multiple-group analysis model.

MODEL	*χ^2^*	*df*	Δ*χ^2^*	*CFI*	*TLI*	*RMSEA*
M3	318.116	122	—	.970	.961	.040
M4	336.330	132	18.214	.969	.963	.040
M5	340.966	135	22.850^*^	.968	.963	.039
M6	341.311	136	23.195	.968	.964	.039
M7	345.165	138	27.049^*^	.968	.964	.039

*χ^2^*: Chi-square statistic; df, Degrees of freedom; Δχ^2^, Change in chi-square; CFI, Comparative Fit Index; TLI, Tucker-Lewis Index; RMSEA, Root Mean Square Error of Approximation; * *p* < 0.05.

M3 is the unconstrained model; M4 is the model of equal measurement coefficients added to M3; M5 is the model of equal structural coefficients added to M4; M6 is the model of equal structural covariance added to M5; and M7 is the model of equal structural residuals added to M6.

## 4 Discussion

This study examined the mediating effect of negative rumination on college students’ interpersonal security and social media dependence and cohort differences between only children and non-only children. The results indicated that interpersonal security may directly or indirectly affect college students’ social media dependence through negative rumination, and the mediating effects differed between the only-child and non-only-child cohorts.

First, our found that interpersonal security had a significant negative predictive role on social media dependence, i.e., the lower the interpersonal security, the higher dependence on social media dependence among college students. This result validates our research hypothesis and is also similar to previous results (33). According to the psychological decompensation hypothesis model, individuals gain psychological security and trust through establishing close interpersonal relationships, but when significant others engage in unreliable behaviors, the sense of security ‘collapses ‘, which then triggers compensatory behaviors to restore the sense of security (34). Social media, as the main medium of internet use, is a common means for individuals to satisfy their socialization needs (35). Internet addiction is a compensatory reaction of adolescents who feel insecure when they get along with others (36) and a lack of interpersonal security is an important factor for individuals to develop social media dependence.

Second, the results of the study showed that interpersonal security was able to negatively predict college students’ social media dependence through the mediating effect of negative rumination, which verified the psychological decompensation hypothesis (15). Empirical studies have confirmed that negative rumination is a mechanism in interpersonal relationships and interpersonal insecurity triggers negative rumination 12. This finding is similar to previous findings that social anxiety predicts negative rumination, namely, people with social anxiety often focus on negative information and think about it repeatedly (37). On the other hand, the negative effects of negative rumination on social media dependence have been supported by several studies (38, 39). For example, previous study showed that people with depression or anxiety are prone to experiencing negative rumination, which leads to their excessive use of cell phones to seek solace (23). In terms of theoretical framework, our results can be explained by a cognitive-behavioral model. According to the cognitive-behavioral model (40), an individual’s stressful life events can be considered as distal causes of social media dependence, whereas rumination can be considered as a proximal cause of social media dependence. Distal factors (i.e., interpersonal security) can influence social media dependence through proximal factors (i.e., negative rumination). In other words, individuals with low interpersonal security may develop self-doubt and negative self-evaluations when they are frustrated in real interpersonal interactions and may constantly ruminate on causes, then activating negative rumination; leading them to rely more on virtual social environment for compensation.

Moreover, our study found significant differences between only children and non-only children in the mediating role of negative rumination between interpersonal security and social media dependence. Negative rumination fully mediated the relationship between interpersonal security and social media dependence for non-only children, while it only partially mediated the relationship for only children. Specifically, the path coefficient of interpersonal security and social media dependence in the only-child group was significantly greater than that in the non-only-child group, which is consistent with the explanation of the feedback effect. Previous study showed that non-only children who have siblings in the family to keep them company develop better social skills to understand others’ emotions, intentions, and psychological states than only children do (41). In interpersonal communication, they have both good peer support and strong social skills (42). However, individuals in only-child families may be more independent. Studies have shown that only children perceive themselves to be significantly less social than others when self-reporting their own social skills (43). Therefore, when frustrated in interpersonal relationships, only children are more likely to rely on social media to alleviate their interpersonal insecurities.

The finding that only children partially mediate the relationship between interpersonal security and social media addiction implies that other factors also play a mediating role. For example, self-esteem has been shown to mediate the relationship between interpersonal security and social media addiction (44). Compared to non-only children, only children have a secure attachment style, which leads to higher self-esteem (45), which would reduce their likelihood of internet addiction. We hypothesized self-esteem may be another mediating factor influencing interpersonal security and online media dependence among only children.

This study has some limitations, including the use of a cross-sectional design and self-report questionnaires. A cross-sectional design makes it difficult to explore causal relationships, and future research could use a longitudinal design to investigate causal links. Furthermore, future research could assess social media dependence levels using objective behavioral indicators to reduce measurement error.

From a psychological education perspective, this study emphasizes the mediating role of negative rumination between interpersonal security and social media dependence. By identifying negative rumination as a key mediator, our findings suggest that interventions targeting rumination reduction could help alleviate social media dependence. Psychological interventions, such as cognitive-behavioral therapy (CBT), could focus on enhancing students’ emotional regulation skills and challenging negative thought patterns to mitigate social media dependence.

Additionally, with changes in China’s family planning policy, the impact of family structure and children’s upbringing environments on individual psychological development may vary. Future research could further explore the role of these cultural and family background factors in social media dependence and investigate how personalized psychological health interventions can be implemented for different groups.

## 5 Conclusion

To summarize, the results showed that a significant interrelationship between interpersonal security, negative rumination, and social media dependence. In addition, the role of negative rumination as a mediator of interpersonal security and social media dependence was supported, and the mediating effects were different between the only-child and non-only-child cohorts. Findings of the study provide a psychological basis for the treatment of social media dependence behavior in college students, with the aim of increasing their interpersonal security and reducing their dependence on social media.

## Data Availability

The raw data supporting the conclusions of this article will be made available by the authors, without undue reservation.
